# Eyes on me: how social media use is associated with urban Chinese adolescents’ concerns about their physical appearance

**DOI:** 10.3389/fpubh.2024.1445090

**Published:** 2024-07-31

**Authors:** Ruining Jin, Tam-Tri Le

**Affiliations:** ^1^Civil, Commercial, and Economic Law School, China University of Political Science and Law, Beijing, China; ^2^Independent Researcher, Ho Chi Minh City, Vietnam

**Keywords:** social media, adolescents, physical appearance, appearance concern, Chinese society

## Abstract

**Introduction:**

Social media usage carries risks of negative impacts on one’s perception of physical attractiveness, especially among adolescents who are developing their self-image. These findings suggest that targeted interventions focusing on attention-seeking behaviors may be effective in addressing appearance-related anxieties among adolescents.

**Methods:**

We employed Bayesian analysis with Markov Chain Monte Carlo algorithms on survey data from 11,926 middle school students in China.

**Findings:**

Our findings indicate that while the amount of daily social media usage does not significantly correlate with appearance concerns, the desire for social media attention through interactions such as liking, commenting, and sharing shows a clear positive association. Female adolescents exhibit a higher degree of concern about their physical appearance compared to males. Additionally, behaviors aimed at improving perceived physical attractiveness, such as exercising and using skin-whitening products, are positively associated with increased appearance concerns.

**Discussion:**

These findings suggest that targeted interventions focusing on attention-seeking behaviors may be effective in addressing appearance-related anxieties among adolescents.

## 1 Introduction

### 1.1 Appearance concern in adolescents

Almost every adult can relate to memories of being a bit “too conscious” of their physical appearance throughout their adolescence. While it is a natural part of growing up and starting to interact with society, an overemphasis on one’s own looks can be psychologically burdensome and distracting for adolescents. Prior to exploring the physiological and psychological transformations that occur throughout puberty, it is crucial to grasp the fundamental ideas that influence how adolescents perceive themselves. The term body image pertains to an individual’s subjective evaluation of their physical appearance, including their thoughts and emotions regarding their body size, shape, and attractiveness (1). Societal beauty ideals, which are established by society, frequently exert an impact on this matter by defining the criteria for physical attractiveness. The range of these values can differ significantly and exert a significant influence on an individual’s self-esteem and pleasure with their body (2). On the other hand, the term self-image encompasses not only the perception of one’s physical appearance but also the beliefs about one’s capabilities, personality traits, and overall value (3). It is the mental picture that an individual holds toward themselves, influenced by both internal self-reflection and external feedback from others. In this regard, self-image perception is the perception of how individuals interpret these aspects of themselves among others during social interactions, which can be critical in their self-esteem and mental wellness (4). Understanding these notions is vital since they have a substantial impact on how adolescents perceive themselves during the key period of puberty, characterized by profound physical and psychological transformations.

During puberty, individuals experience notable physiological changes, which prompt a process of adaptation wherein heightened levels of self-awareness and vulnerability to social evaluation might occur as a result of their self-assessment of physical attractiveness (5). Due to their limited experience and cognitive capability, adolescents are more prone to internalizing unattainable beauty standards, as they lack the critical ability to scrutinize messages and feedback from the social infosphere (6). Failed or unsuccessful adaptation attempts can lead to feelings of discomfort or self-doubt regarding their developing physical attributes, as they strive to conform to societal norms and seek validation from their peers (5). These feelings are also known as social appearance anxiety (SAA)—a specific type of social anxiety defined by the fear of being negatively evaluated because of one’s appearance. Individuals diagnosed with SAA commonly experience concerns regarding their physical appearance, encompassing aspects such as weight, skin condition, hair quality, and dental health (7, 8). Symptoms of this disorder include pronounced shyness, avoidance of social engagements, and withdrawal from exploratory activities (9, 10).

From a gender perspective, females are more likely to experience negative social evaluation fears related to their physical appearance and, therefore have a higher propensity to suffer from SAA compared with their male counterparts (11–13). In terms of coping styles, when facing SAA or other mental distress, females tend to have higher rates of rumination, social support seeking, and emotional expression, whereas males demonstrate higher rates of substance use, denial, and humor when coping with stress or distress (14).

One of the common behaviors in response to concerns about one’s appearance is physical exercise. Regular exercise has been shown to produce changes in physical appearance that people often aspire to, including increased muscle mass, reduced body fat, and a greater physique in general (15, 16). Exercise provides adolescents with a sense of control over altering their appearance in a healthy manner. Besides direct physical changes, there are also psychological benefits from exercising. Being physically active can help adolescents feel better about their body image and manage insecurities over perceived unattractiveness (17, 18). Exercise that builds muscle tone and cardiovascular fitness can boost adolescents’ confidence and self-esteem by allowing them to appreciate what their body can do versus how it looks (19). Sports and activities that emphasize teamwork over appearance help redirect focus toward collaboration and away from unrealistic beauty ideals (20). Namely, adolescents with strong social support and a sense of belonging can gain from participating in sports provide a buffer against appearance-based rejection or teasing (20, 21). Therefore, establishing healthy fitness habits and realistic goals allows adolescents to have greater ownership over their body’s abilities and take pride in physical competencies (22, 23). In this way, physical exercise can be an adaptive coping strategy for appearance anxiety (19).

Skincare practice is another common behavior for improving physical appearance but has some unique characteristics in the context of modern East Asian society. Due to sociohistorical factors such as the perception of “*一白遮百丑*” (*a white complexion can hide several flaws*), and the low social hierarchy and status associated with darker skin colors (association with lower-class physical labor), Chinese society has strong favoritism for light skin color (24–26). For instance, a study conducted by (27) revealed that a significant majority of Chinese female college students (over 70%) expressed dissatisfaction with their skin complexion and expressed a desire to achieve a lighter skin tone. Furthermore, the study discovered that approximately 60% of the participants had utilized skin-lightening products as a means to achieve this desired outcome. To mitigate skin color anxiety, many Chinese females choose to use skin whitening products – a historical practice that can be traced back to the Tang Dynasty when women used ground pearl powders for skin whitening (28). Nowadays, using skin whitening products has become a common practice for Chinese women on a daily basis (27).

### 1.2 Effects of social media use on self-image perception

Social media usage can be a significant stressor when it comes to adolescents’ SAA, as it may negatively impact adolescents’ self-esteem, self-image, and a distorted perception of beauty standards. One study on adolescents’ Instagram use suggested that exposure to idealized images of peers on Instagram led to lower self-esteem and body satisfaction among adolescent girls (29). Similarly, a study on college students found Instagram usage was associated with increased bodily and appearance-based social comparison and lower self-esteem (30). In this regard, the more frequent use of social media, the greater the risk of increased body image concerns and poorer psychological adjustment for adolescents (31). Expanding on the previous discourse regarding the body image and self-perception of adolescents, a recent study offers a more profound understanding of the impact of social media in this area (32). In this study, it emphasizes the complex connection between the usage of social media and worries about body image, with a specific focus on how feelings of shame about one’s body and participation in social media influence the formation of self-image among adolescents. This is consistent with another study (33), which examined the wider effects of social media on eating disorders and body image problems. These study highlights the important influence of social media on how adolescents view themselves and their concerns about weight. In addition, another study (34) on the locus of control in women who have received plastic surgery provides a distinct viewpoint on the impact of external factors, such as social media, on self-image and body image perception. Moreover, one more study (35) enhances our comprehension by investigating the impact of “fitspiration” photos on social media, which can engender detrimental body image comparisons among young individuals. One qualitative research (36) investigates positive body image encounters, offering a counterpoint to the frequently adverse effects of social media, and emphasizes the significance of familial and educational support in cultivating a wholesome self-perception. These studies emphasize the intricate relationship between the use of social media and how adolescents perceive their physical appearance. This highlights the importance of adopting a detailed strategy for comprehending and tackling these issues.

In terms of Chinese adolescents’ social media use, one study indicated that Chinese adolescents averaged 2.95 h per day on electronic media use, and the use time of urban adolescents with higher familial socioeconomic status would be even longer (37). While there are a few recent studies suggesting that social media use is associated with negative mental health outcomes for Chinese youth (38–41), there are also studies suggesting that social support and scientific knowledge from digital spaces can be beneficial to one’s mental and physical health (42, 43). Speaking of gender differences, one recent study highlighted that although both male and female Chinese adolescents predominantly use WeChat and QQ as their preferred social media platforms and view social media as neither facilitating nor inhibiting their peer relationships and academic learning, females are more likely to interact socially and share personal moments and engage more with social topics, whereas males focus more on gaming and sports (44).

From a Social Comparison perspective, humans have a natural tendency to evaluate their abilities by comparing themselves to others. In other words, people tend to seek external references when objective information about themselves is unavailable (45). There are two main types of social comparisons: upward comparisons and downward comparisons. Through upward and downward comparisons, individuals are more likely to compare themselves to those who could offer meaningful comparisons. In this case, when adolescents use social media, they can be exposed to idealized images, objectified bodies, and unrealistic beauty standards (29). Therefore, when they view popular/viral posts from others, they might end up in envy and feel mental distress. In a study regarding strangers’ positive Instagram posts, it was found that those who frequently compare themselves with others would report lower positive affect if they had viewed positive strangers’ posts than if they had viewed neutral or no posts (46).

On a separate note, Self-objectification theory was developed in 1997 by psychologists Fredrickson and Roberts (47). The theory examines the process of girls and women internalizing an observer’s perspective of their physical selves due to continual sexual objectification and body evaluation in society, which leads to constant self-monitoring of appearance over competence. Social media platforms emphasize image and video sharing, propagating idealized, sexualized depictions of people’s bodies through influencers. Therefore, features like Likes, Comments, and Reposts invite external evaluation of one’s appearance, which might lead to possible self-objectification. In addition, these features can also be linked to financial incentives, as marketing firms normalize the use of social media influencers in their marketing campaigns (48, 49). So, users would be further incentivized to view themselves from an observer’s perspective and constantly monitor how they look online to receive better feedback. As a result, the use of social media perpetuates the objectification of people (especially women) and leads users to overvalue their physical attractiveness at the expense of authentic self-representation.

In addition, according to the Fear of Negative Evaluation theory, some individuals have an excessive amount of fear when they are judged negatively by others. Therefore, they are apprehensive about others’ opinions, avoid evaluative situations, and are sensitive to rejection (50). In social media, they may excessively worry about posting self-related content that could be criticized, as negative comments or lack of likes/follows can be anxiety-provoking to young people affected by the fear of negative evaluation.

Moreover, social media interaction patterns and instant gratification can be attention-grabbing and addicting. Therefore, an increased engagement with social media activities can increase the focus on adolescents’ objectified selves, disengaging from other personal focuses. It has been observed that individuals who excessively consume addiction-related content tend to exhibit a notably heightened level of “cue reactivity” (51). This heightened reactivity is associated with an increased desire for and inclination to engage in addictive behaviors when compared to neurotypical individuals who do not consume such content (52). One study’s findings (53) are an example indicating the negative relationship between Instagram social media addiction and self-esteem in high school students.

Psychological pathways of information exchanges in modern social media interactions are complex and novel. For examining this emerging multifaceted matter, the aforementioned major related theories are used as guidance for the logical conceptualization of analyses as well as corresponding interpretations in the present study. While previous studies have focused on certain Chinese social media platforms which target particular social groups, exploring its impact on adolescents’ mental health conditions (54, 55), little has been known about social media use in general and how it would impact urban adolescents’ mental health conditions. Thus, considering the background information and foundational theories above, we aim to examine how Chinese urban adolescents’ social media use in general may affect the degree of their focus on ones’ physical appearance. Specifically, we examine if the quantity of daily social media usage as well as a desire to receive attention through social media platforms’ interactions increase the focus of concern on one’s own look. Additionally, we examine if females have a higher degree of appearance concern compared to males. Possible associations with common behaviors related to attempting to improve one’s physical appearance including exercising and using skin-whitening products (specific to China’s social context) are also examined.

The research objective of the present study is as follows: examining how the degree of concern about one’s appearance may be associated with the following factors: social media usage quantity, desire for attention on social media, physical exercise frequency, gender, and skin-whitening product usage.

## 2 Methodology

### 2.1 Materials and variables

Survey data was collected from 11,926 students from 11 middle schools in Jiangsu Province, China between April 8, 2023, and May 29, 2023. Overall, middle school students from eight public schools and three international schools were sampled in Jiangsu Province, China. Four public schools and two international schools are located in Suzhou, and four public schools and one international school are located in Nanjing. Both Suzhou and Nanjing are economically affluent cities, as Suzhou is ranked 6th and Nanjing is ranked 10th in terms of total city Gross Domestic Product (GDP) in China (56). Therefore, participants in this study were generally from urban middle/upper-middle class families in China. In total, 4,349 (36.47%) participants self-identified as being biologically male, whereas 7,577 (63.53%) identified themselves as biologically female. The convenient sampling approach was employed. Students’ ages range from 13 to 18 years old (studying in grade 7 to grade 12). Although previous scales have been developed to measure social media use, SAA and skin color discrimination/anxiety (57–60), they are largely based on Western and non-Chinese contexts, which would not fit well in the current study context. To identify the appropriate content for the questionnaire, pilot interviews with students were conducted in advance, including 48 participants with each session lasting for 15–30 min.

Data collection was approved by the Institutional Review Board of the China University of Political Science and Law. The rights and wellbeing of all participants were protected. Because emails are less popular in China compared with how they are in Western countries as a formal communication tool, and WeChat as a mega app is also widely used as a work communication tool in China, therefore it is not practical to gain informed consent forms from parents and students via emails or in a face-to-face manner given the large sample size; instead, the researchers met with all head teachers in advance to explain the purpose and procedures of the research, including the importance and procedures of the informed consent form, then the researchers shared the informed consent form with the head teachers via WeChat. Then the head teachers sent the informed consent form to the group chat of the school-parent WeChat Groups. Headteachers of each class collected the informed consent forms with parents’ and students’ signatures and sent them back to the researchers. Collecting informed consent through WeChat is in alignment with standard research procedures in China.

In this study, we use ordinal measurement for daily social media usage. Riehm et al. (61) study indicated that more than 30 min of social media use per day is associated with an increased risk of internalizing problems and comorbid internalizing and externalizing problems in adolescents. So and Chin (62) study found that 2 h and 30 min is a threshold for high-risk internet addiction. Per Chinese clinical diagnostic criteria for internet addiction (63), “If a person uses the internet for more than 6 h a day and meets certain conditions continuously for over 3 months, it is considered as Internet Addiction according to clinical standards, and internet addiction is classified as a mental disorder.” Therefore, we set the values at below 30 min, 30 min to 2 h and 30 min, 2 h and 30 min to 6 h, and more than 6 h. Also, the use of ordinal value is not uncommon among similar studies, namely, in the Chinese government-led survey on public school students *2021 National Research Report on Internet Use Among Minors* also used a similar ordinal measurement, “(1) <30 min; (2) 30 min to 1 h; (3) 1 h to 2 h; (4) 2–3 h; (5) 3–5 h; (6) More than 5 h” in its data collection (64). Likewise, Riehm et al. (61) also used an ordinal value of self-reported time spent on social media during a typical day (none, ≤30 min, >30 min to ≤3 h, >3 h to ≤6 h, and > 6 h).

Table 1 presents the selected variables from the dataset to be used in the present study’s statistical analysis.

**Table 1:** Variable description.

Variable name	Meaning	Variable type	Value
*Appearance*	Focusing on concerns about one’s appearance over other things	Ordinal	1. Totally disagree 2. Somewhat disagree 3. Somewhat agree 4. Totally agree
*SocialMedia*	Daily social media usage	Ordinal	1. Less than half an hour 2. Half an hour–2.5 h 3. 2.5–6 h 4. Over 6 h
*GetAttention*	Hoping to get attention on social media	Ordinal	1. Strongly disagree 2. Somewhat disagree 3. Somewhat agree 4. Strongly agree
*Exercise*	Physical exercise frequency	Ordinal	1. Never 2. Occasionally 3. Once a week 4. At least thrice a week
*Female*	Being a female	Binary	0. No 1. Yes
*Whitening*	Currently using skin-whitening product(s)	Binary	0. No 1. Yes

The variable *Appearance* represents the degree of focus on concerns about one’s appearance. It comes the rating one’s agreement with the statement “I care more about how I look, rather than the things around me,” where responses are measured on a 4-point Likert scale from “Totally disagree” (1) to “Totally agree” (4). This question belongs to the section about social appearance anxiety. The variable *SocialMedia* represents the degree of self-reported daily social media usage. The variable *GetAttention* represents the degree of agreement to the statement “I hope my social media posts can receive more likes, attention, comments, and shares,” where responses are measured on a 4-point Likert scale ranging from negative to positive. The variable *Exercise* represents the self-reported frequency of weekly physical exercise. The binary variable *Female* was from a demographic information question about the participants’ biological sex. The binary variable *Whitening* represents the status of whether a participant was currently using skin-whitening products.

### 2.2 Analysis procedure

In the present study, we employ Bayesian analysis with aided Markov Chain Monte Carlo (MCMC) algorithms. The procedures of model construction, statistical analysis, and result presentation were conducted following the protocol for MCMC-aided Bayesian analysis in social research (65). MCMC algorithms helped generate a large number of simulated data points based on the original data, which increases the accuracy of the estimated posterior results. The normalization transformation of data distributions also helped minimize skewness within the collected data, providing sufficient effective data points for the inference. Technical validation procedures for the analytical model are presented in the paragraph below. Multi-regression is conducted using the following formula, where $\mu_{i}$ is the mean value of participant i ’s appearance concern (outcome variable *Appearance*) with posterior estimations in the form of normal distribution.


$$\begin{array}{l} \mu_{i}=\beta_{0}+\beta_{\mathrm{SocialMedia}}*\mathrm{ocialMedi}a_{i}+\beta_{\mathrm{GetAttention}}*\mathrm{GetAttentio}n_{i}+\\ \;\beta_{\mathrm{Exercise}}*\mathrm{Exercis}e_{i}+\beta_{\mathrm{Female}}*\mathrm{Femal}e_{i}+\beta_{\mathrm{Whitening}}*\mathrm{Whitenin}g_{i} \end{array}$$

The analytical model has an intercept $\beta_{0}$ and coefficients $\beta_{\mathrm{LightSkin}}$ , $\beta_{\mathrm{NotAttractive}}$ , $\beta_{\mathrm{Evaluated}}$ , $\beta_{\mathrm{Bullied}}$ , $\beta_{\mathrm{Favoritism}}$ , and $\beta_{\mathrm{Female}}$ . The respective measured values for participant i are $\mathrm{SocialMedi}a_{i}$ , $\mathrm{GetAttentio}n_{i}$ , $\mathrm{Exercis}e_{i}$ , $\mathrm{Femal}e_{i}$ , and $\mathrm{Whitenin}g_{i}$ .

In Bayesian analysis, all properties are treated probabilistically including unknown parameters. Result interpretation is based on the highest occurrence probabilities of parameters regarding their posterior distributions, which can aid assessment accuracy in psychological research (66–69). The analytical model was constructed following the parsimonious model principle to increase predictive power, especially when conducting research with an exploratory nature. To ensure the reliability of the Bayesian inference in the analysis, the model’s goodness of fit is checked using Pareto-smoothed importance sampling leave-one-out (PSIS-LOO) diagnostics (70, 71). Here, if there are *k* values exceeding 0.7, there are problematic observations that can influence the MCMC processes. If all *k* values are below 0.5, the simulated data fit well with real data. The Markov chains’ convergence is checked using indicators of the effective sample size (*n_eff*) and the Gelman-Rubin shrink factor (*Rhat*). Here, *n_eff* values should be over 1,000 (72), and *Rhat* values should be 1 (73, 74). The analysis procedure and data interpretation followed the protocol for MCMC-aided Bayesian regression analysis in social sciences (65, 75). The analysis is conducted using the *bayesvl* package in R (76), with R version 4.2.0, uninformative priors. This package has good visualization capability, which is beneficial for presenting validation checking and posterior distribution ranges. Uninformative priors were used to avoid subjective influence toward the inference, considering the exploratory manner of this analysis. MCMC setup is 5,000 iterations (including 2000 warm-up iterations), and four chains.

## 3 Results

**Figure 1:**
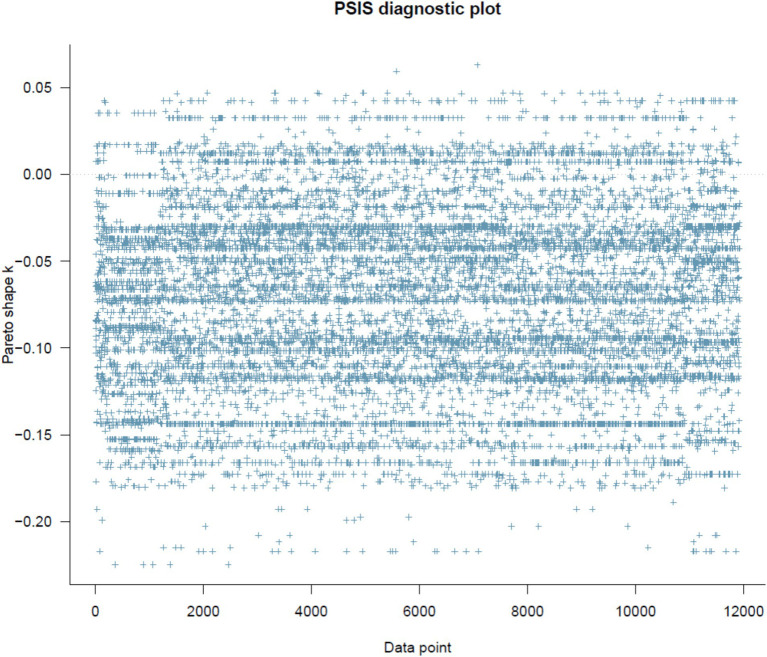
PSIS diagnostic plot.

Figure 1 shows the result of the PSIS diagnosis. All *k* values are way below the threshold of 0.5, indicating high goodness of fit.

Table 2 summarizes the statistical results of the analysis. The values of n_eff are over 1,000 and Rhat equal 1 for all parameters, signaling good reliability of the results.

**Table 2:** Model 1’s simulated posteriors.

Parameters	Mean (M)	Standard deviation (S)	n_eff	Rhat
*Constant*	0.93	0.06	4,707	1
*SocialMedia*	0.01	0.01	6,246	1
*GetAttention*	0.42	0.01	9,946	1
*Exercise*	0.11	0.01	6,423	1
*Female*	0.09	0.02	10,003	1
*Whitening*	0.13	0.02	10,001	1

**Figure 2:**
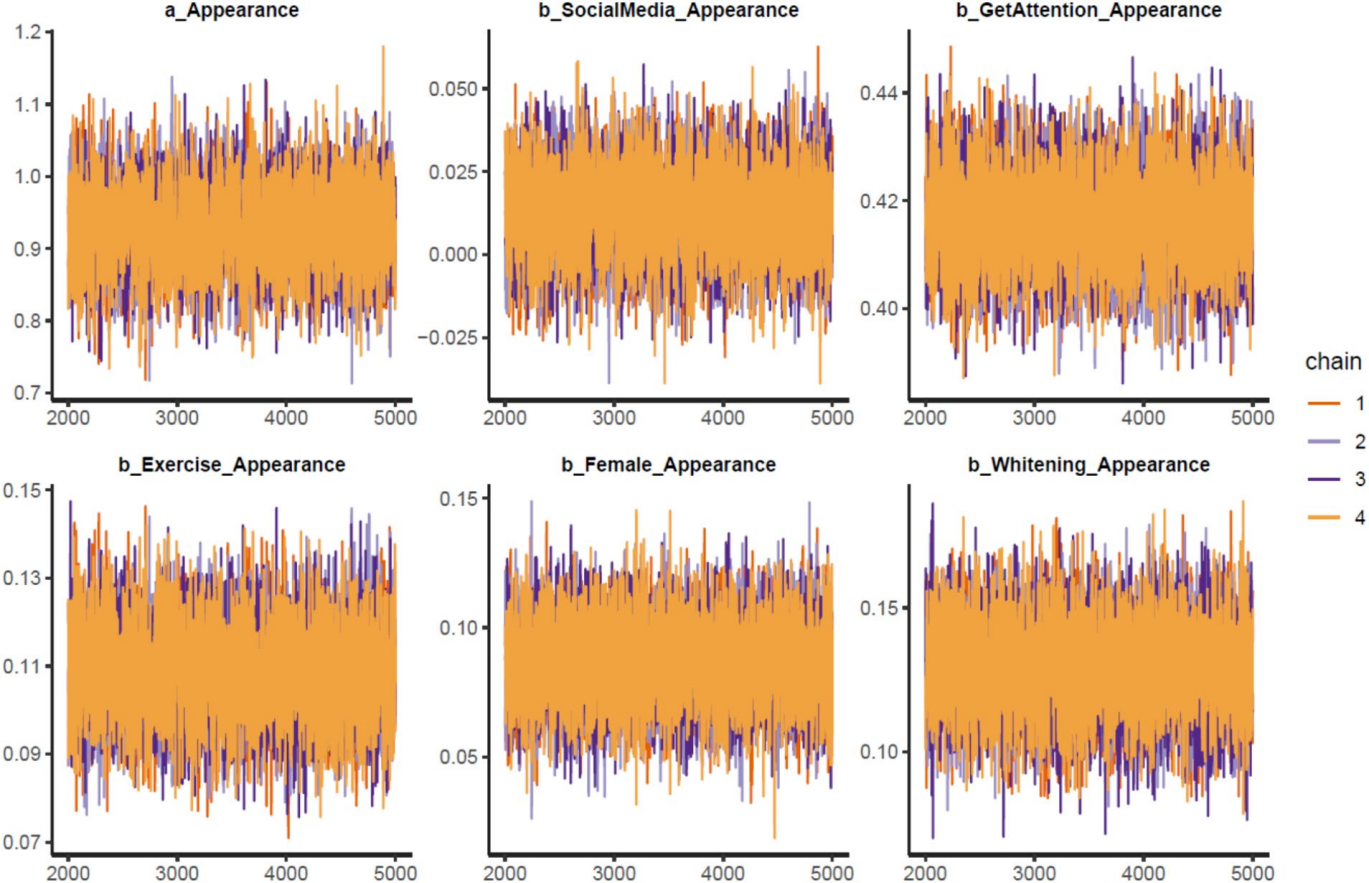
Trace plots.

Markov chain convergence is also visually presented in the trace plots (see Figure 2). After the warm-up period (from the 2000th iteration onward), the chains fluctuate around equilibriums for all parameters and do not have any significant deviation.

**Figure 3:**
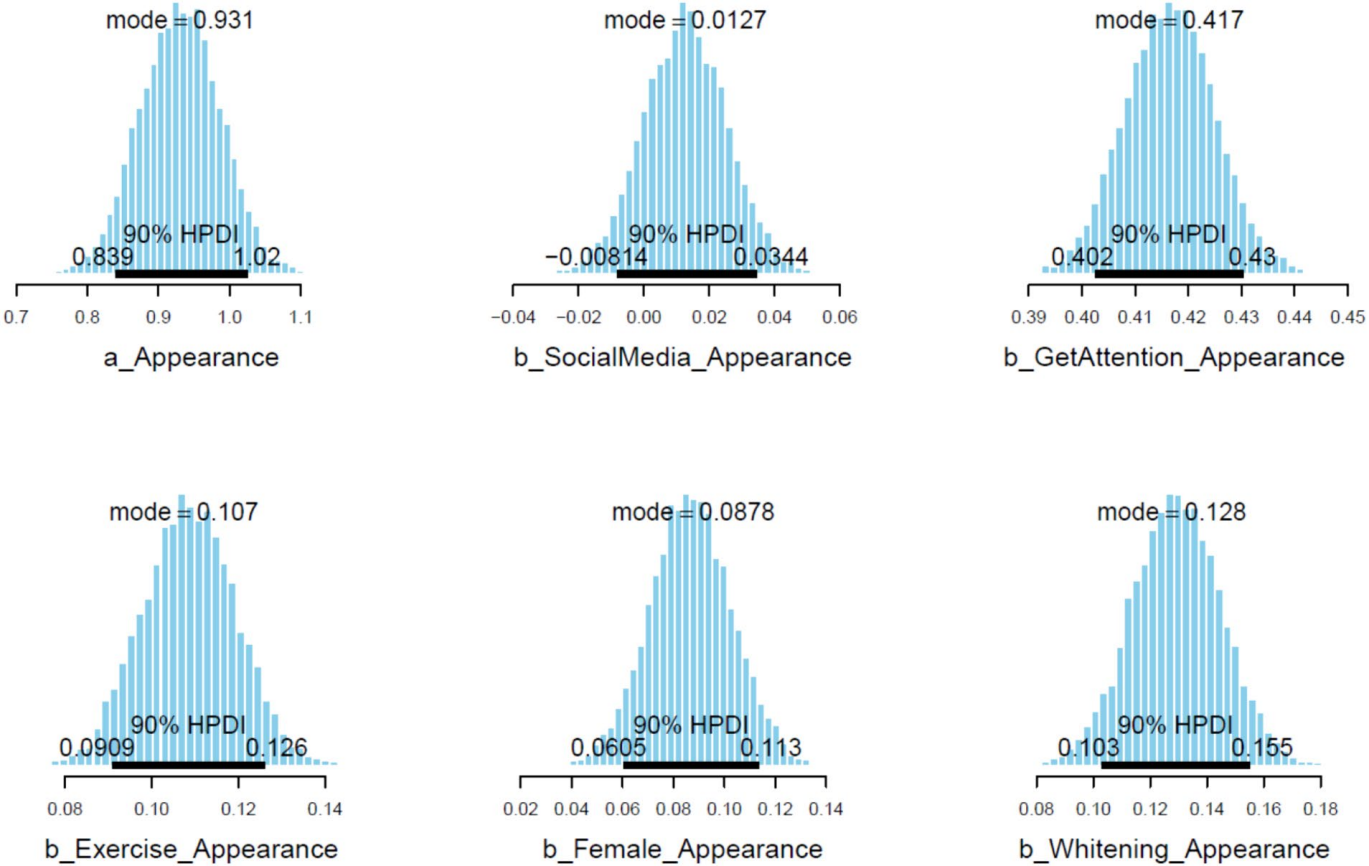
Posterior distributions within 90% highest posterior density intervals (HPDIs).

As shown through the posterior coefficient in Table 2, *SocialMedia* does not have any significant association with *Appearance* ( $M_{\mathrm{SocialMedia}}$ = 0.01 and $S_{\mathrm{SocialMedia}}$ = 0.01). However, *GetAttention* has a clear positive association with *Appearance* ( $M_{\mathrm{GetAttention}}$ = 0.42 and $S_{\mathrm{GetAttention}}$ = 0.01). This effect has a relatively high magnitude compared to other factors. *Exercise*, *Female*, and *Whitening* were all found to have positive associations with *Appearance* ( $M_{\mathrm{Exercise}}$ = 0.11 and $S_{\mathrm{Exercise}}$ = 0.01, $M_{\mathrm{Female}}$ = 0.09 and $S_{\mathrm{Female}}$ = 0.02, $M_{\mathrm{Whitening}}$ = 0.13 and $S_{\mathrm{Whitening}}$ = 0.02). The standard deviation values after 5,000 iterations are small because the original sample size is relatively large. The posterior distributions of the coefficients within 90% highest posterior density intervals (HPDIs) are visualized in Figure 3. For *GetAttention*, *Exercise*, *Female*, and *Whitening*, the distributions lie completely on the positive side, indicating the high reliability of the found positive associations.

## 4 Discussion

In our analysis, we did not find a correlation between focus on one’s appearance and the amount of time one uses social media daily. However, we found a clear and significant association between focus on one’s appearance and one’s desire to receive attention on social media through interactions such as liking, commenting, and sharing. Female adolescents were found to have a higher degree of focus on their physical appearance compared to males, which aligns with former research suggesting that females are more prone to concern about physical attractiveness than males (11) and females tend to respond to emotional concerns regarding physical attractiveness with a higher degree of rumination (14). Additionally, our findings also show that certain behaviors involving attempts to improve perceived physical appearance – specifically: exercising and using skin-whitening products – are positively associated with a higher degree of focus on one’s appearance.

The result that social media usage time alone does not affect concerns about one’s appearance is not surprising, given the neutral nature of the activity in general. Social media are tools of information exchange. For example, WeChat is one of the most well-used digital applications in China, deeply embedded in the daily lives and social interactions of users across age groups and demographics (77, 78). Its feature – WeChat Moment (similar to Instagram) – has been reported to increase upward and downward social comparisons among students and young adults (79, 80). It also integrates WeChat Official Accounts (similar to information update features in Twitter or LinkedIn), which present information published by credible sources, including central and local governments, schools, and other organizations. This was suggested to help disseminate vital information during a public health crisis (81) as well as for educational purposes (82). The quantity of social media exposure in terms of usage time, thus, does not necessarily link to specific directions of impacts.

Directional influences from using social media platforms are due to the desires, purposes, attitudes, and vulnerabilities of users. Thus, the clear effect of one’s desire to receive attention on social media through interactions follows this line of logic. This result provides a specific angle to look at how social media usage may have negative impacts on one’s self-image, especially physical appearance, as suggested in former studies (29–31). The desire to gain liking, commenting, and sharing quantity of one’s social media posts, when it comes to content related to one’s physical appearance (e.g., photos and videos showing oneself) can lead to self-objectification, which intensifies the focus on physical appearance over other aspects of oneself (47).

In social media, in addition to the acquisition or generation of subjective values through social comparisons, users may also reinforce a desire to receive attention. This reinforcement is further motivated by the way social media systems design sponsorships, affiliate marketing, and merchandising (49, 83). Because physical attractiveness likely promotes more media interactions such as likes, comments, and reposts (84, 85), users might put a heavier focus on their appearance to compete for attention in social media. Furthermore, norms established by influencers promoting unrealistic beauty standards on social media also contribute to the association between social attention and one’s attractive appearance (27, 86). In the attempts to gain more attention on social media, users try to improve self-marketing related to their physical appearance, which exacerbates the impacts of self-objectification.

Additionally, fear of negative evaluation (here, through social media interactions) also increases the sensitivity toward concerns about self-perceptions, including physical appearance (50). Overall, a stronger emphasis in the mind on one’s own looks is reinforced. Adapting behavior in the hope of improving one’s physique is expressed through a higher frequency of exercising, considering the direct impacts of exercising on the body (15, 16, 87, 88). Furthermore, in Chinese society where lighter skin color is collectively perceived as a beauty standard (24–26), skincare practices in the form of skin-whitening product usage are also employed as an adapting behavior for improving one’s looks.

The information coming from social media that may affect one’s appearance concern can be looked at in terms of several “dimensions”: direction, intensity, and compatibility. The direction of information determines whether the information enhances or diminishes one’s self-image. Regardless of the direction as attached subjective meaning, the intensity of such information determines its power of possible impact. This is represented by the amount of exposure through the total time of social media usage. However, only those who have motivation on appearance-based interactions are compatible with the exposed information on such platforms. In other words, adolescents hoping to get attention online are more sensitive to information that relates to and potentially impacts their self-image. Ideations involving physical exercise and using skincare products may also be in a feedback loop with increasing the intensity of thinking and rumination about self-image.

From the findings of this study, there are some implications for policymaking on adolescent psychological wellbeing. Social media usage, on its own, is simply utilizing digital platforms for social interaction. The risk factor for distorted self-perceptions is not the act of using social media, but the accompanied attitudes while using such platforms/services. Thus, considering the common practices of using manipulative tactics and algorithms of social media companies, following the precautionary principle, the government should have measures to prevent major harmful psychological effects from happening. How toxic degrees of desire for attention are formed should be well-understood rather than simply attempting to restrict personal usage time. In the context of Chinese society, the government issues certain top-down measures on this matter. However, it is also important to carefully consider whether such measures may be too inflexible (focusing on superficial restrictions and hindering the digital market’s development) or too invasive to users’ digital privacy. A recent example is presented below.

China’s State Council recently announced the *Regulations on the Protection of Minors Online,* which went into effect on January 1, 2024 (89). In this regulation, particular guidance was offered to protect minors from online addiction and inappropriate content. Namely, article 43 requires online service providers (online games, live streaming, audio and video, and social media) to “adhere to the principles of integration, user-friendliness, practicality, and effectiveness” in accordance with the characteristics of minors of different ages using their services, and offer a special mode that “controls usage time, duration, functions, and content,” as well as “conspicuous and convenient reminding features to guardians regarding minor users’ time management, parental management, and consumption management to assist guardians in their supervisory roles.” Article 45 explicitly addresses that online service providers “must take measures to prevent and resist the value prioritizations of attention and click rate,” and “must not set up online communities, groups, or topics themed around support fundraising, ranking votes, or manipulating statistics and comments,” nor should they induce minors to participate in similar activities. The regulations aim to create a safer online environment for minors, balancing their use of digital services with their overall wellbeing. However, the policy’s appropriateness and practical effectiveness should be carefully assessed on real data in the near future.

Aside from regulating social media companies, better monitoring programs should be established on social media platforms to oversee and discourage influencers from tapping into the attention economy using psychological manipulation techniques related to physical attractiveness. Proper mental health counseling and support at school, family, and local community also help adolescents develop a healthy attitude toward their self-image in regard to social comparison. Likewise, it is beneficial to help adolescents realize that behaviors such as exercising, and skincare practices should be driven by overall wellbeing improvement motivations rather than solely due to social anxiety about one’s physical appearance.

There are some limitations in the present study. Firstly, the convenient sampling only consists of participants from two economically affluent cities in Jiangsu Province. Students from lower socioeconomic backgrounds such as those who live in rural areas or inner continental areas of China may have different psychological patterns. Future studies may compare the findings in distinctly different economic zones. The updating manner of Bayesian inference is helpful when attempting to update the found patterns with newly available data. Secondly, in our data, biological sex was reported and self-identified genders were not included. While LGBTQ+ groups constitute a relatively small proportion of the Chinese adolescent population, their psychological patterns regarding physical appearance and corresponding social anxiety may have unique patterns. Future studies may examine deeper on this aspect. Thirdly, the subtle influences of ideological directionality on collective psychological expression were not within the scope of this study. The conduct of this study was not confined to any specific interpretations by certain authorized Chinese government departments regarding accessibility and adverse effects on minors in the context of internet use. Such strict top-down regulatory approaches may intersect with broader considerations highlighted in the Universal Declaration of Human Rights (UDHR), particularly regarding the importance of information transparency and the right of individuals to access information. Future scholarly inquiry regarding Chinese digital environments may explore the nuanced balance between ensuring the safety of minors in digital spaces and upholding human rights principles, including information accessibility and transparency. Fourth, the current study examined social media use in general. However, there are Chinese social media platforms targeting particular social groups such as Xiaohongshu and its target audience of young urban females (90, 91). Future research can explore the effects of such platforms on their specific targeted populations.

## Data availability statement

The datasets presented in this study can be found in online repositories. The names of the repository/repositories and accession number(s) can be found in the article/supplementary material.

## Ethics statement

This study has been approved by the Institutional Review Board (IRB) of the China University of Political Science and Law. The IRB ensures that all ethical standards have been met and that the rights, welfare, and wellbeing of all participants have been fully considered and protected. All authors read and approved the final version of the manuscript. The studies were conducted in accordance with the local legislation and institutional requirements. Written informed consent for participation in this study was provided by the participants’ legal guardians/next of kin.

## Author contributions

RJ: Conceptualization, Writing – original draft, Writing – review & editing. T-TL: Conceptualization, Supervision, Writing – review & editing.
